# Predictors of Death Rate during the COVID-19 Pandemic

**DOI:** 10.3390/healthcare8030339

**Published:** 2020-09-14

**Authors:** Ian Feinhandler, Benjamin Cilento, Brad Beauvais, Jordan Harrop, Lawrence Fulton

**Affiliations:** 1 Department of Geography and Political Sciences, Front Range Community College, Longmont, CO 80501, USA; ian.feinhandler@frontrange.edu; 2 Memorial Hermann Hospital, Houston, TX 77024, USA; bencilento@me.com; 3 School of Health Administration, Texas State University, San Marcos, TX 78666, USA; bmb230@txstate.edu; 4 Acushnet Corporation, Boston, MA 02743, USA; jordan_harrop@acushnetgolf.com

**Keywords:** COVID-19, geospatial regression, health disparities, public health

## Abstract

Coronavirus (COVID-19) is a potentially fatal viral infection. This study investigates geography, demography, socioeconomics, health conditions, hospital characteristics, and politics as potential explanatory variables for death rates at the state and county levels. Data from the Centers for Disease Control and Prevention, the Census Bureau, Centers for Medicare and Medicaid, Definitive Healthcare, and USAfacts.org were used to evaluate regression models. Yearly pneumonia and flu death rates (state level, 2014–2018) were evaluated as a function of the governors’ political party using a repeated measures analysis. At the state and county level, spatial regression models were evaluated. At the county level, we discovered a statistically significant model that included geography, population density, racial and ethnic status, three health status variables along with a political factor. A state level analysis identified health status, minority status, and the interaction between governors’ parties and health status as important variables. The political factor, however, did not appear in a subsequent analysis of 2014–2018 pneumonia and flu death rates. The pathogenesis of COVID-19 has a greater and disproportionate effect within racial and ethnic minority groups, and the political influence on the reporting of COVID-19 mortality was statistically relevant at the county level and as an interaction term only at the state level.

## 1. Introduction

Severe acute respiratory syndrome coronavirus 2 (SARS-CoV-2) is the etiologic agent of the coronavirus (COVID-19) pandemic. As of 31 August 2020, the associated death toll in the United States is reported to have surpassed 180,000 [1], the highest of any country in raw numbers but equivalent to many other developed countries when adjusted for population [2]. The proper recognition and remediation of the disease are pressing concerns and each will likely be subject to debate in the months prior to the 2020 presidential election [3,4]. However, there is some concern surrounding the veracity of the data and factors contributing to COVID-19 deaths. Media outlets provide daily updates on the number of cases and deaths but draw this information from data collection and reporting agencies that have adjusted their methods over time [5]. The resulting inconsistencies have led to charges of underreporting [6,7] and overreporting [8,9], and have contributed to the politicization of the pandemic.

COVID-19 data inconsistencies and potential political bias in data reporting can have significant implications. If the data that politicians rely on are faulty, subsequent policies may harm public health, the economy, and other aspects of society. Testing differences, false positives, false negatives, and other factors that likely differ from state-to-state and county-to-county make the underlying official deaths with COVID-19 reports somewhat suspect; however, this study leverages the official data used by the Centers for Disease Control and Prevention (CDC). There are several county level studies about COVID-19 available from recent research. Badr et al. (2020) evaluated mobility patterns and COVID-19 transmission [10]. This study provided county level spread data but did not focus on deaths. Scannell et al. (2020) demonstrated racial disparities at the county level for COVID-19 cases and deaths [11]. Cases, unfortunately, suffer from severe measurement problems, as will be discussed. Ives and Bozzuto (2020) analyzed county level estimates of R0, the basic reproduction number for COVID-19 [12]. Altiera et al. (2020) estimated county level deaths used in estimating required medical supplies [13]. Two articles consider political factors—Flanders et al. (2020) assessed voter turnout as related to COVID-19 [14], and Makridis and Rothwell (2020) evaluated the effects of political polarization but not in terms of death rates [15]. We found no other paper that addresses death rate disparities by including a political variable. Thus, given the novel nature of the virus and its progression and the known inconsistencies in the reported data, we sought to gain a deeper understanding of the factors that contribute to reported deaths from COVID-19.

### 1.1. Research Questions

We investigated three research questions. First, what attributes of geography, demography, population density, economy, population health, hospital characteristics, and politics might explain the deaths per 100,000 (death rate) at the county level as of 31 August 2020? Second, did COVID-19 death rates at the state level differ based upon governor party affiliation after accounting for other relevant variables? As a control for our second line of inquiry, we also examined whether variation existed in previous flu/pneumonia death rates (2014–2018) based upon the governor’s party affiliation.

### 1.2. Significance and Motivation

To our knowledge, this research is the first to evaluate COVID-19 using combined data from multiple areas covering demographic, socioeconomic, health system, population health, and political factors using a spatial regression. It is also the first study to evaluate the effects of state and county political affiliation on COVID-19 death rates. The motivation behind this study is to address the media promulgation of explanatory factors that may or may not be scientifically verifiable (e.g., population density and political factors), particularly when placed in the context of other known factors established at the individual unit of analysis (e.g., race).

## 2. Methods

### 2.1. Sample Sizes and Data Sets

Sample sizes for the research questions were 3116 (county), 51 (states plus Washington D.C.), and 250 (50 states by 5 years). The dependent variable was the death rate per 100,000 population. Cumulative COVID-19 deaths were obtained from USAfacts.org [1] for 31 August 2020. Flu data were from the Centers for Disease Control and Prevention, CDC, from 2014–2018 [16]. Definitive healthcare data provided descriptive hospital-related information [17]. Population and demographic data were from the Census Bureau [18]. The Centers for Medicare and Medicaid Services (CMS) provided the source for relevant patient morbidity proportions by state and county [19]. Geographic variables in the analysis included the shapefiles from the Census Bureau’s state and county Tiger Files [20].

### 2.2. Variables

The race and ethnicity variables included the proportion of African Americans, Native Americans, Asians, and Hispanics. The proportion of Caucasians was omitted due to collinearity considerations. Population density (population per square kilometer), and the proportion of people aged 65 and older served as additional control variables, although we anticipated (correctly) that the former might not enter the model, particularly when geospatial effects were considered. Economic variables included the median household income and unemployment. Population health status variables included the population proportions with chronic obstructive pulmonary disease (COPD), heart failure, diabetes, obesity, and cancer, all of which have been identified by the CDC as risk factors at the individual level [21], as well as other health-related variables including smoking, obesity, alcohol abuse, Alzheimer’s Disease, asthma, atrial fibrillation, depression, drug abuse, HIV, hepatitis B, and stroke. Health system capability variables included the number of acute beds in the county or state and the average case-mix index in the county or state. The case-mix index, or CMI, adjusts inpatients based on severity, with 1.0 being the “typical” visit and higher average numbers meaning more acute visits than would be expected.

### 2.3. Reasons for Variable Inclusion and Expected Effects

Geography was included as a known predictor of COVID-19 [22]. Similarly, demographics [23,24], population density [25], proportion of people aged 65 and older [21], economic considerations [26], population health status (comorbidities) [27], and political considerations [28] are also known as hypothetical factors that affect infection and death rates, although the reasons for the associations between individual variables and death rates are not fully understood [24]. We include hospital system characteristics to account for the possibility that lack of resources increase death rates [29].

Based on these research studies, we surmise that higher population densities might initially be associated with higher death rates, but that the effects of including spatial models will remove these effects. Increases in population density may place individuals at an increased risk of exposure. A better economic status (e.g., lower poverty rates) should result in better access to healthcare systems and thus lower death rates. Poverty, for example, results in reduced compliance with COVID-19 protocols [30]. Higher rates of comorbidities (e.g., health status) are likely to be associated with higher death rates [31]. An improved hospital capability and lower patient severity might reduce death rates [29]. Finally, there is much speculation that political considerations are influencing both death rates and the reporting of death rates, where Democratically affiliated geographies are anticipated to have higher death rates [32].

### 2.4. Transformations

Quantitative variables were standardized. At the state level of analysis, the small number of observations (51) necessitated data reduction. We used the first three principal components of all health status variables to proxy the effects of population health. These three components accounted for 75% of the variability of the original 19 variables.

### 2.5. Models

We evaluated least absolute shrinkage and selection (lasso) models [33] to generate a subset of variables associated with deaths per 100,000 using adaptive *p*-values as presented by Lockhart et al. [34] and implemented in the covTest package [35] in R [36]. The adaptive *p*-values address Lindley’s paradox, which often requires that the significance level changes as sample size increases [37]. We also used 10-fold cross-validation to evaluate *R*^2^ and the root mean squared error (RMSE) along with associated standard deviations (SDs). Appendix A Table A1 is a list of the independent variables evaluated.

After fitting the Ordinary Least Squares (OLSs) model and constrained models, we repeated the same process to fit geospatial models. Specifically, we used a residual analysis to fit appropriate geospatial models with all of the variables and the subset suggested by lasso. Moran’s I and Lagrangian multiplier diagnostics were used to recommend the appropriate geospatial model to be fitted (none, spatial lag, or spatial error).

We also investigated reporting differences that might exist for flu and pneumonia deaths at the state level. Using a repeated measures analysis, we modeled the logarithm of flu and pneumonia deaths as a function of year and governor party. All analyses were performed in R Statistical Software [36].

## 3. Results

All code is available for replication. County level R code (updated through 31 August 2020) is available online [38]. State level code (also updated through 31 August) and influenza analyses are available online as well [39]. 

### 3.1. Descriptive Statistics

Table 1 summarizes the descriptive statistics at the county level of analysis. At the county level (as of 31 August 2020), the mean COVID-19 death rate is 33.84. The mean county population was 9% African American, 2% Native American, 9% Hispanic, 1% Asian, and 20% aged 65 and over. Population density, income, and unemployment averages were 106.45 per square kilometer, USD 53,000 per county person and 4% per county, respectively. The largest comorbidity proportion average was adult obesity (32.85%), and the mean number of acute beds was 215 with a median of 35. The average CMI was 1.06 with a median of 1.17. Sixteen percent of counties voted for the Democratic candidate in 2016.

**Figure 1:**
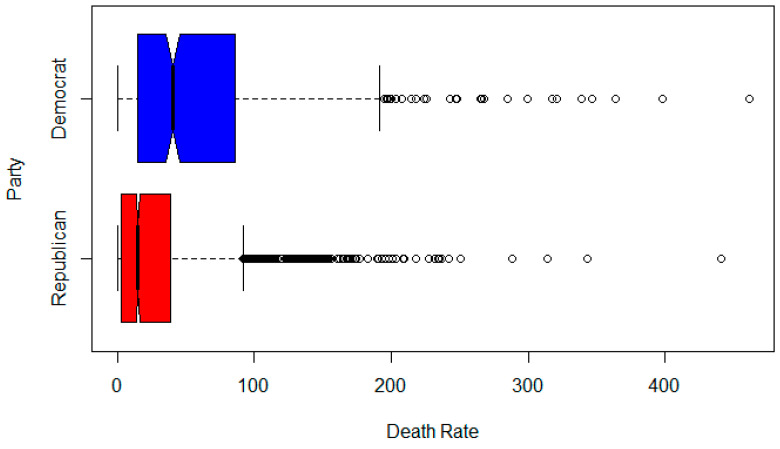
Boxplots of Republican versus Democratic county death rates per 100,000.

Figure 1 is a notched boxplot of the death rate of Democratic counties versus Republican counties. The notch indicates the statistical significance (median test) at the α = 0.05 level. There appears to be a statistically significant difference between the two group’s death rates per 100,000 people.

**Figure 2:**
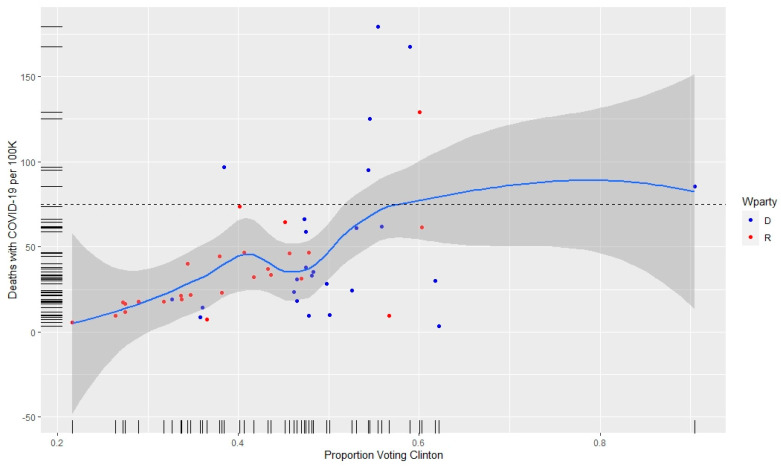
Coronavirus (COVID-19) death rates per 100,000 (*y* axis) as a function of proportion voting for Clinton in 2016 (*x* axis) and the current party of the governor as a red or blue dot.

Figure 2 provides a scatterplot of the proportion voting Democrat in a state versus the deaths per 100,000 with symbols showing which states voted for Clinton versus Trump. Seven states have at least 75 deaths per 100,000. Of those states, six voted for Clinton. The red and blue dots indicate the current party of the state governor.

Table 2 presents a county level summary of the association between 2016 presidential election results, population density, and deaths from COVID-19. The population density is higher for counties that voted Democratic (116.2 versus 23.5), as are the death rates (71.0 versus 36.8).

At the state level (Table 3), descriptive statistics are provided for variables considered for the final model. The deaths per 100,000 for COVID-19 were 45.74 versus flu deaths of 15.10 per 100 K. The proportions of African Americans, Native Americans, Hispanics, and people 65 years of age (and older) were 11.27%, 1.62%, 12.01%, and 16.39%, respectively. Unemployment in 2019 averaged 3.62%, and about 49% of the states had Democratic governors.

### 3.2. COVID-19 Death Analysis, County

The four models estimated for the county analysis are depicted in Table 4. Column 1 shows the estimates for the full OLS model. The lasso model is shown in column 2. The geospatial models (full and reduced based on residual analysis) are shown in columns 3 and 4.

#### 3.2.1. Ordinary Least Squares (OLSs) Full Model

**Figure 3:**
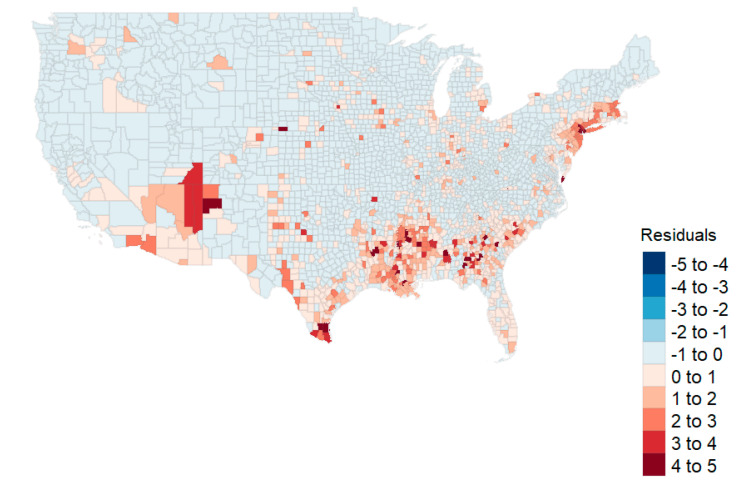
Residual plot from the Ordinary Least Squares (OLSs) model shows clusters in the Northeast and Southwest.

The full OLS model (“OLS Full”) is depicted in the first columns of Table 4. The highest variance inflation factor (VIF) was 3.706 (poverty). The model accounted for 37.9% of the variability (*R*^2^). No statistically significant effect for the county’s winning party was apparent in the first model evaluation (*p* = 0.242). Figure 3 shows the map of the residuals for the full OLS model, indicating that some spatial autocorrelation exists in the northeast and the southwest areas of the country. Moran’s I analysis suggested a geospatial correlation as well (I = 0.253, *p* < 0.001).

#### 3.2.2. Lasso Model

The best-tuned lasso model RMSE was 0.800 with a standard deviation (SD) of 0.045. The predicted *R*^2^ was 0.352 with a standard deviation of 0.028. The lasso model (“Lasso”, Table 4) using adaptive *p*-values identified likely predictors such as race, ethnicity, and three health status variables (Alzheimer’s Disease, COPD, and diabetes). The model produced a similar *R*^2^ as the unconstrained model (*R*^2^ = 0.374). This constrained regression model also suggested that the political factor (winning party) should be considered as a potential explanatory variable (*p* = 0.089). Residual patterns were similar to Figure 2, and Moran’s I was statistically significant, indicative of a spatial correlation (I = 0.265, *p* < 0.001). The Lagrange multiplier diagnostics again recommended a lag model.

#### 3.2.3. Generalized Spatial Two-Stage Least Squares Model, All Variables

A generalized spatial two-stage least squares model (GS2SLS) [40] was used on the full set of independent variables. This model (“GIS Full”, Table 4) identified that geospatial location was important for explaining the death rate (*ρ* = 0.634). Variables in the model again included the political factor (winning party). The residuals from the geospatial model no longer exhibited an autocorrelation (Moran’s I = −0.098, *p* = 0.980).

#### 3.2.4. Generalized Spatial Two-Stage Least Squares Model, Lasso Variables

A final reduced model included the variables identified by the lasso as part of a geospatial lag model. This final model (Table 4, “GIS Reduced”) also included the political factor, and again, the residuals were stable based on a Monte Carlo simulation of Moran’s I (I = −0.070, *p* = 0.980). For interpretability, the unscaled geospatial model is shown in Table 5.

In Table 5, the reduced geospatial analysis with unscaled variables suggests that geospatial effects, population density, ethnicity and race, unemployment, three health status variables, and the winning party are important in explaining the death rates per 100,000. Native American, Hispanic, and/or African American proportions are associated with a 42.728, 23.226, and 52.703 increase in deaths per 100,000 individuals, respectively. County political leaning based on the 2016 presidential election is associated with an increase of 4.503 deaths per 100,000 individuals (dichotomously coded variable). Moran’s I was not significant (I = −0.070, *p* = 0.9804).

An important result is that while we evaluated population density, its standardized effect size was almost zero (0.003) when other factors were considered. This county level analysis is congruent with Pew Research findings that death rates are higher in Democratic-led counties [32]. This study suggests that the racial/ethnic composition and geographic relationships with the outbreak are important considerations along with political considerations. Further, we note that the results of the spatial analysis are similar to those of the nonspatial analysis. The implication may be that our county level models are robust.

### 3.3. COVID-19 Death Analysis, State

Given the results of the political analysis at the county level, we further evaluated political leadership at the state level, examining a subset of variables found from the county level analysis. Since only 51 observations were available, the analysis was restricted to the minority proportion in the state (1-proportion Caucasian only), the first three principal components of health status variables (accounting for 75% of the variability), population density, unemployment, the governor’s party, and plurality [20]. Plurality was dichotomously coded with 0 = plurality (the 2016 voting consensus matching the governor’s party) and 1 = no plurality (voting block different from the governor’s party). We also surmised that there might exist an interaction effect between the governors’ party and health status and modeled the interaction terms accordingly. Death rates were mapped, and states in the Northeast (New Jersey, New York, Massachusetts, and Connecticut) had higher death rates than other areas of the country. These states were omitted in a secondary analysis to ensure that the results found were not due strictly to outliers.

**Figure 4:**
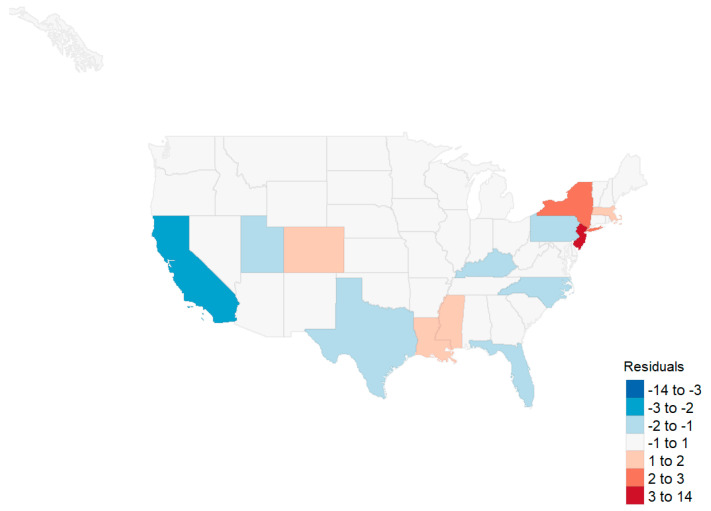
Residuals, state level initial analysis.

An OLSs model using the aforementioned variables captured 66% of the variability with the highest VIF of 3.24. Statistically significant variables included the minority population, all three health status principal components, and the interaction term between the governor’s party and the first principal component (the linear combination representing the primary comorbidities of the population). Moran’s I did not suggest that a spatial model was required at the state level (I = 0.060, *p* = 0.162). A map of the residuals is shown in Figure 4. When removing the outliers of New Jersey, New York, Massachusetts, and Connecticut, minority status was the remaining statistically significant variable. Health status and the governor’s party interaction with health status fell out of the model (Table 6).

### 3.4. Flu Death Analysis, State

As a final analysis, we investigated death rates from past influenza outbreaks and governors’ parties, a proxy for party politics. Since we found an effect at the county level and an interaction effect at the state level, we wanted to see if this was constant over time based on another respiratory disease. To investigate, we ran a repeated measures (by state) analysis of variance on the log-transformed death rate for 2014–2018. The model identified no effects associated with the governor party affiliation (F_(1, 244)_ = 1.531, *p* = 0.217), only the reporting year (F_(4, 244)_ = 2.382, *p* = 0.040).

## 4. Discussion

### 4.1. Summary of Results

In this study, we first ran a county level analysis for death rates based on geographical, socioeconomic, health status, health capability, and political groupings. Our investigations were reduced to two full OLS models and two geospatial models. From our analysis, it was clear that geospatial models with lags were preferred to the OLS models. Further, the reduced GIS model using only variables identified from lasso produced nearly the same *R*^2^ as the full GIS model (0.500 versus 0.507, respectively). Thus, the reduced model performs nearly as well as the full model in estimating county death rates. In that model, we see significant geospatial effects (*ρ*), as well as those associated with population density, race, and the winning party in the 2016 election. The estimate for Democratic counties (untransformed) was 4.503 deaths per 100,000.

For the state level analysis, we found effects associated with the proportion minority, three principal components associated with health status variables, and the interaction between the governor’s party and the first health status variable. However, when removing the four states with the highest death rates (New Jersey, New York, Massachusetts, and Connecticut), we found that the only predictive variable was the minority proportion in the state. Further, an analysis of influenza death rates showed no effect associated with political party.

### 4.2. Population Density Effects

Population density has been identified as a predictive factor in disease progression [41,42]. A superficial examination of county level data indicates that a relationship might exist between population density and death rate from COVID-19 (see Table 2). Consistent with prior analysis [43,44], Table 2 also shows urban areas tended to vote Democrat in the 2016 presidential election. Due to these associations, media outlets have presented the urban–rural divide as a viable explanation for the difference in death rates between counties that voted Democrat in 2016, and those that voted Republican [45,46]. This divide has also provided an explanation for the divergent response to the disease based on party affiliation. For example, Democrats are more concerned about COVID-19 than Republicans, and are more likely to wear a facemask and practice other forms of social distancing [28,47,48]. However, the effect size of population density at the county level is negligible when other factors are considered. For example, in the reduced GIS model for counties, the standardized coefficient is only 0.051. Population density does not appear as a significant variable in the state level models. The failure of population density to provide a more significant explanation for deaths from COVID-19 has been one of the surprising results from our analysis.

### 4.3. Race and Ethnicity/Minority Effects

At the county level, our study confirms the findings of numerous researchers pertaining to healthcare disparities in the United States, particularly with respect to Native American, Hispanic, and African American populations [49,50,51]. We found an increase in the percentage of these populations to be associated with an increase in mortality from COVID-19 at the county and state levels of analysis. McLaren (2020) attributes this difference to disparities in education, occupation, and commuting patterns [51]. The causes of disparity, however, are not explained by the covariates in this study (see Carl, 2020 [52]). Although we did not include these factors in our analysis, we did find the mortality disparities do not appear to be attributable to differences in unemployment rates or household income. Our county findings suggest that there are healthcare disparities in the United States, but may also be indicative of a pathogenesis of COVID-19 that has a greater and disproportionate effect within these three racial groups [53,54]. At the state level, increases in minority population proportions were also associated with increases in death rates per 100,000.

### 4.4. Health Status Effects

At the state level, health status (measured by three principal components and the interaction between the governor’s party and the first principal component) was a predictor for the *n* = 51 state observations. These health status effects disappeared after removing the four outlier states from the model. Thus, it would appear that minority status is the predominant predictor such that increases in the proportion of minorities are associated with increases in deaths per 100,000.

### 4.5. Unemployment Effects

At the county level (and consistent with prior research), unemployment characteristics were identified as having a significant association with COVID-19-related deaths [44,45]. While this association is clear, its causation is not. It is possible that unemployment increases exposure to the disease; for example, cost-cutting might lead to increased use of public transportation. It is possible that unemployment increases vulnerability to the disease through elevated stress levels and poor nutrition. The unemployed may also be left without access to healthcare, which increases mortality from disease. However, it is also possible that unemployment increases the incidence of deaths of despair (deaths due to drug, alcohol, and suicide), and that these excess deaths (defined by the CDC as the difference between the observed numbers of deaths and expected number of deaths in a specific time period) [55] are being reported as COVID-related. For example, on 13 April 2020, New York City added more than 3700 people to the COVID-19 death total – people who were presumed to have died of the coronavirus but had never tested positive [56,57]. Without a positive test, it is impossible to know if these additional deaths—at the time, 37% of the city’s total—were actually COVID-related, were deaths of despair, or were due to other causes. 

Periods of economic downturn have long been found to be associated with declines in health status and higher suicide rates compared with periods of relative prosperity [46,47,48]. Recent research has found a 17% increase in drug overdose nationally during April and May 2020 [58]. Compounding the problem, there are indications that a prolonged and overly restrictive COVID response is deepening an already deleterious economic cycle, the result of which is increased unemployment [49]. As unemployment increases, so does the mortality rate either directly or indirectly from the disease. In short, extended efforts to eradicate the disease may cause additional harmful secondary and tertiary effects that may be worse than the disease itself.

### 4.6. Political Party Effect

The influence of politics on the reporting of COVID-19 mortality was a significant finding in our analysis. County level Democratic affiliation was significantly associated with increased COVID-19 deaths, even after controlling for factors such as population density. To the best of our knowledge, this is the first time that population density and urbanization are used as controls when evaluating death rates between Democratic and Republican states.

In past years, the CDC retrospectively tabulated the number of flu-associated illnesses, hospitalizations, and deaths—a process that takes up to two years to generate an estimate. The process relies on estimation modeling in and out of hospitals based on behavioral algorithms [59]. The CDC never relies solely on death certificate data because it recognizes that there is never large-scale testing and that the clinicians do not routinely list influenza data on death certificates if the patient died of pneumonia, heart failure, or deteriorating lung disease. According to the CDC, this leads to significant underreporting of deaths due to flu every year [59].

On 20 February 2020, the CDC published guidelines for the diagnosis and mandatory reporting of COVID-19 for any patients evaluated with “COVID related” illnesses. This applied to all healthcare practitioners and included a comprehensive set of instructions and codes to document any relationship to COVID-19 on the death certificates [60]. This represents a significant change in reporting of the disease and consequently the inclusion on the death certificate. Three separate additional guidelines put out in March and April affirmed these measures. In addition, the new CDC guidance stated that: “In cases where a definite diagnosis of COVID–19 cannot be made, but it is suspected or likely, it is acceptable to report COVID–19 on a death certificate as ‘probable’ or ‘presumed’” [60]. This change introduced significant potential variations in the tabulation of COVID-19 death tolls.

At approximately the same time, the Centers for Medicare and Medicaid Services (CMS) authorized an additional 20% reimbursement for patients carrying a diagnosis of COVID-19 pursuant to Sections 3710 and 3711 of the CARES Act [61]. These changes created a financial incentive for hospitals to classify patients as positive for COVID-19. Importantly, at the time these measures were introduced, the dominant model used by policy-makers—based on Ferguson et al. [62]—predicted an exceptionally high mortality rate [63]. By late March, more accurate estimates predicted a mortality rate well below original expectations [64]. This should have triggered a policy reversal from the CDC and CMS, but no changes were noted. In short, in the politically charged landscape of 2020, the CDC’s new way of collecting data, combined with CMS’ monetary incentives, may have resulted in the overreporting of COVID-19 deaths. The introduction of these two new sources of reporting bias makes historical comparisons unreliable at best. Without reliable data, it is difficult to effectively fight a pandemic. This conundrum associated with the reliability of data on COVID-related deaths highlights the need for objective and uniform standards for case identification and data collection.

## 5. Conclusions

During our analysis, we evaluated the data that pointed toward political interference in the reporting of COVID-related deaths. As of 31 August 2020, it is clear that the national death rate from COVID-19 is higher than from other flu pandemics, but the increase in the reported death rate in states with Democratic governors has been greater than the increase in states with Republican governors. Much more research in the area of politicization of medical reporting is needed, particularly given the political climate of the United States.

One of the major limitations of this study is that the associated methods are unable to estimate causality. Any variable found to be unimportant in this analysis might have its effects mediated out by others. The coefficient estimates are associated with the model built, and the associated *p*-values suggest the importance of that model. A second important limitation is that this analysis is current only as of 31 August 2020. The analysis will continue to change as the pandemic peaks and subsides.

Future research should supplement this analysis by investigating whether states with contested gubernatorial elections (e.g., those with ballot purges, an issue that is becoming more commonplace [65]) report higher mortality rates than those with normal elections. Additional research should focus on time series models as well as simulations to generate forecasts with the external regressors identified by this research.

## Figures and Tables

**Table 1:** County level descriptive statistics.

Variable (*n* = 3116 Counties)	Mean	SD	Median	Minimum	Maximum
Population in 2020	105,237	334,733.38	26,163.00	169	10,039,107
Population Density (persons per km^2^)	106.45	696.94	17.50	0	27755
Native American %	1.57%	6.48%	0.30%	0.00%	89.60%
Hispanic %	9.30%	13.84%	4.10%	0.00%	99.10%
African American %	8.99%	14.51%	2.20%	0.00%	87.40%
Asian %	1.31%	2.59%	0.60%	0.00%	43.08%
% 65 or older	19.79%	4.76%	19.40%	4.90%	58.20%
Unemployment % (2019)	3.96%	1.39%	3.70%	0.70%	18.30%
Household Income USD (2018) ^+^	USD 52,714.43	USD 13,851.63	USD 50,531.00	USD 25,385.00	USD 140,382.00
Poverty %	15.17%	6.11%	14.10%	2.60%	54.00%
Smoke %	17.44%	3.56%	16.95%	5.91%	41.49%
Adult Obesity %	32.85%	5.43%	33.10%	12.40%	57.70%
Alcohol Abuse %	2.24%	1.01%	2.21%	0.00%	10.36%
Alzheimer’s %	10.17%	2.18%	10.15%	0.00%	25.02%
Asthma %	4.31%	1.34%	4.35%	0.00%	11.64%
Atrial Fibrillation %	8.03%	1.61%	8.12%	0.00%	17.50%
Cancer %	7.41%	1.40%	7.43%	0.00%	12.10%
Kidney % *	22.85%	4.51%	22.94%	0.00%	51.45%
COPD %	12.81%	3.77%	12.44%	0.00%	32.15%
Depression %	17.44%	3.57%	17.48%	0.00%	35.87%
Diabetes %	26.93%	5.09%	27.11%	0.00%	49.62%
Drug Abuse %	3.14%	1.83%	2.93%	0.00%	16.70%
HIV %	0.11%	0.25%	0.00%	0.00%	4.51%
Heart Failure %	14.39%	3.28%	14.15%	0.00%	33.75%
Hepatitis B %	0.47%	0.42%	0.49%	0.00%	4.10%
Hyperlipidemia % **	38.04%	8.80%	39.35%	0.00%	67.55%
Hypertension %	56.51%	8.77%	58.30%	0.00%	74.95%
Ischemia % ***	26.84%	5.44%	26.68%	0.00%	46.91%
Stroke %	3.32%	1.09%	3.35%	0.00%	9.46%
Number of Acute Beds	215	720.47	35	0	19274
Case Mix Index	1.061	0.587	1.170	0.000	2.710
2016 Winning Party (1 = Democrat)	0.158	0.364	0.000	0.000	1.000
Deaths/100K	34.030	46.753	17.753	0.000	461.156

^+^ collinear with poverty, r = −0.771, * collinear with diabetes, r = 0.78, ** collinear with hypertension, r = 0.80, *** collinear with heart failure and hypertension, r = 0.71 for both.

**Table 2:** Population density and COVID-19 deaths by 2016 electoral outcome (31 August 2020).

Candidate	Counties Won	Avg. Density	Deaths	Death Rate
Clinton	491	116.2	126,554	71.0
Trump	2625	23.5	55,157	36.8
Total	3116	41.5	181,711	55.4

**Table 3:** State level descriptive statistics.

Variables (*n* = 51)	Mean	SD	Median	Minimum	Maximum
% African American	11.27%	10.72%	7.50%	0.40%	46.90%
% Native American	1.62%	2.87%	0.50%	0.20%	14.40%
% Hispanic	12.01%	10.31%	9.52%	1.43%	49.09%
% 65 and over	16.39%	1.99%	16.40%	11.10%	20.60%
% Unemployment	3.62%	0.82%	3.50%	2.40%	6.10%
% Democratic Governor	49.02%	50.49%	0.00%	0.00%	100.00%
COVID-19 Deaths/100 K	45.74	39.58	32.95	5.01	179.53
Flu Deaths/100 K	15.10	3.76	14.65	7.00	29.60

**Table 4:** Model results (scaled variables).

Variable	OLS Full	*p*	Lasso	*Adaptive p*	GIS Full	*p*	GIS Reduced	*p*
*R* ^2^ *(Predicted R* ^2^ *for Lasso)*	*0.377*		*0.352 +/- 0.800*		*0.507*		*0.500*	
Rho					0.634	<0.001	0.589	<0.001
Intercept	0.000	0.014	0.000	NA	−0.004	0.732	−0.004	<0.001
Pop. Density	0.163	0.017	0.138	0.038	0.066	<0.001	0.051	<0.001
% Native American	0.090	0.018	0.057	0.038	0.070	<0.001	0.059	<0.001
% Hispanic	0.133	0.022	0.132	<0.001	0.082	<0.001	0.071	<0.001
% Black	0.408	0.029	0.369	<0.001	0.178	<0.001	0.169	<0.001
% Asian	0.008	0.019			−0.009	0.581		
% 65 and older	0.022	0.019			0.022	0.182		
% Unemployed	0.079	0.018	0.075	0.007	0.052	0.001	0.062	<0.001
Poverty	0.018	0.027			0.012	0.621		
% Smoke	−0.061	0.026			−0.006	0.815		
% Adult Obesity	−0.045	0.019			0.006	0.721		
% Alcohol	0.041	0.020			0.024	0.170		
% Alzheimer’s	0.112	0.021	0.149	<0.001	0.073	<0.001	0.097	<0.001
% Asthma	−0.049	0.020			−0.022	0.217		
% Atrial Fib.	0.017	0.021			0.011	0.563		
% Cancer	−0.010	0.020			−0.016	0.379		
% COPD	−0.074	0.027	−0.104	<0.001	−0.047	0.048	−0.053	0.006
% Depression	0.036	0.023			0.043	0.034		
% Diabetes	0.183	0.027	0.162	<0.001	0.078	0.001	0.079	<0.001
% Drug Abuse	−0.027	0.022			−0.033	0.096		
% HIV	−0.074	0.021			−0.047	0.011		
% Heart Failure	−0.027	0.021			−0.009	0.636		
% Hepatitis B	−0.048	0.021			−0.031	0.095		
% Stroke	0.092	0.022			0.026	0.182		
Number of Acute Beds	−0.006	0.018			0.009	0.565		
Case Mix Index	0.038	0.017			0.045	0.004		
Winning Party	0.029	0.019	0.024	0.089	0.046	0.007	0.032	0.033

**Table 5:** Unscaled geospatial model.

Variable	Estimate	*p*
Rho	0.598	<0.001
(Intercept)	−35.350	<0.001
Population Density	0.003	0.001
% Native American	42.728	<0.001
% Hispanic	23.226	<0.001
% African American/Black	52.703	<0.001
Unemployment Rate	2.112	<0.001
Alzheimer’s Disease	2.077	<0.001
Chronic Obstructive Pulmonary Disease (COPD)	−0.664	0.005
Diabetes	0.716	<0.001
Winning Party, 2016 Election (1 = Democrat)	4.503	0.021

**Table 6:** Results of the regression analyses for the state models.

Variable	OLS Full	*p*	OLS without State Outliers	*p*
R^2^	0.655		0.304	
(Intercept)	−0.007	0.940	0.007	0.961
% Minority	−0.231	0.083	0.421	0.070
Plurality	0.049	0.627	0.078	0.609
Governor’s Party	−0.056	0.609	−0.260	0.137
Unemployment	0.188	0.174	0.159	0.437
% in Poverty	0.198	0.243	−0.270	0.273
Population Density	−0.258	0.116	−0.013	0.959
Health PC1	0.201	0.000	−0.074	0.272
Health PC2	0.388	0.000	0.005	0.977
Health PC3	−0.213	0.029	0.145	0.263
Governor’s Party × Health PC1	0.084	0.027	0.053	0.332

## Appendix A

**Table A1:** Independent Variables Considered in the Analysis.

Description	Source	Description	Source
State FIPS Code	USA Facts	% Hypertension	CMS
State Name	USA Facts	% Ischemic Heart Disease	CMS
County Name	USA Facts	% Stroke	CMS
County FIPS Code	USA Facts	Civilian Labor Force, February 2020	BLS
Population 2020	USA Facts	Employed, February 2020	BLS
Land Area, square kilometers	CB	Unemployed, February 2020	BLS
People per sq. kilometer	Calculated	Percent Unemployed, February 2020	BLS
Urban–Rural Classification	NCHS	Civilian Labor Force, 2019	USDA ERS
% < Poverty Line	USDA ERS	Employed, 2019	USDA ERS
% for Clinton in 2016	MIT	Unemployed, 2019	USDA ERS
Winning Party in 2016	MIT	% Unemployed, 2019	USDA ERS
% Below Poverty Line, 2018	USDA ERS	MHI, 2018	USDA ERS
% Smokers	RWF	Population ≥ 65, 2019	CB
% Adult Obesity	RWF	% age 65 and over, 2019	CB
% Abusing Alcohol	CMS	Median Age, 2019	CB
% Alzheimer’s	CMS	Total Population, 2018	IPUMS
% Asthma	CMS	Racial Data	IPUMS
% Atrial Fibrillation	CMS	# Hospital Physicians	DHC
% Cancer	CMS	# Acute Care Beds	DHC
% Chronic Kidney Disease	CMS	# Intensive Care Beds	DHC
% COPD	CMS	# Staffed Beds	DHC
% Depression	CMS	# Discharges	DHC
% Diabetes	CMS	Sum Average Daily Census	DHC
% Drug Abuse	CMS	Hospital average length of stay	DHC
% HIV	CMS	Average market concentration index	DHC
% Heart Failure	CMS	Average hospital case mix index	DHC
% Hepatitis B or C	CMS	Geographic shape files	CB
% Hyperlipidemia	CMS		

# = Number, CB = Census Bureau [18], NCHS = National Center for Health Statistics [66], USDA ERS = United States Department of Agriculture Economic Research Service [67], MIT = MIT Election Lab [68], RWF = Robert Woods Foundation County Health Rankings and Roadmaps [69], CMS = Centers for Medicare & Medicaid Services [19], BLS = Bureau of Labor Statistics [70], IPUMS = Integrated Public Use Microdata Series [71], DHC = Definitive Healthcare [17].
